# 2-Methyl-1-phenyl-1*H*-indole-3-carbo­nitrile

**DOI:** 10.1107/S1600536811039250

**Published:** 2011-10-08

**Authors:** Kun Yang, Pei-Fan Li, Yan Liu, Zhi-Zhong Fang

**Affiliations:** a Teaching & Research Center, Tianjin Medical University, Tianjin 300070, People’s Republic of China; b Pharmacy Department, Tianjin Medical College, Tianjin 300222, People’s Republic of China

## Abstract

In the title compound, C_16_H_12_N_2_, the dihedral angle between the indole ring system and the pendant phenyl ring is 64.92 (5)°. The crystal packing features aromatic π–π stacking [centroid–centroid separation = 3.9504 (9) Å] and C—H⋯π inter­actions.

## Related literature

For the synthesis of the title compound, see: Du *et al.* (2006 ▶). For its precursor, see: Jin *et al.* (2009 ▶). For related structures, see: Yang *et al.* (2011 ▶); Yan & Qi (2011*a* ▶,*b* ▶).
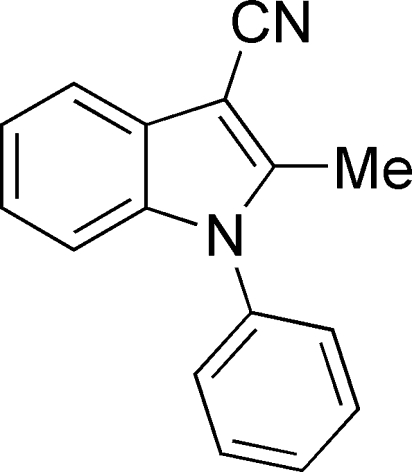


## Experimental

### 

#### Crystal data

C_16_H_12_N_2_
*M* *_r_* = 232.28
Triclinic, 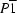

*a* = 6.3610 (5) Å
*b* = 9.497 (1) Å
*c* = 11.0210 (12) Å
α = 65.97 (2)°
β = 80.52 (2)°
γ = 88.13 (2)°
*V* = 599.34 (14) Å^3^
*Z* = 2
Mo *K*α radiation
μ = 0.08 mm^−1^
*T* = 113 K
0.26 × 0.24 × 0.06 mm

#### Data collection

Rigaku Saturn724 CCD diffractometer
Absorption correction: multi-scan (*CrystalClear-SM Expert*; Rigaku, 2009 ▶) *T*_min_ = 0.980, *T*_max_ = 0.995
11425 measured reflections
3494 independent reflections
2309 reflections with *I* > 2σ(*I*)
*R*_int_ = 0.040

#### Refinement

*R*[*F* ^2^ > 2σ(*F* ^2^)] = 0.044
*wR*(*F* ^2^) = 0.120
*S* = 0.98
3494 reflections
164 parameters
H-atom parameters constrained
Δρ_max_ = 0.45 e Å^−3^
Δρ_min_ = −0.29 e Å^−3^

### 

Data collection: *CrystalClear-SM Expert* (Rigaku 2009 ▶); cell refinement: *CrystalClear-SM Expert*; data reduction: *CrystalClear-SM Expert*; program(s) used to solve structure: *SHELXS97* (Sheldrick, 2008 ▶); program(s) used to refine structure: *SHELXL97* (Sheldrick, 2008 ▶); molecular graphics: *CrystalStructure* (Rigaku, 2009 ▶); software used to prepare material for publication: *CrystalStructure*.

## Supplementary Material

Crystal structure: contains datablock(s) global, I. DOI: 10.1107/S1600536811039250/hb6411sup1.cif

Structure factors: contains datablock(s) I. DOI: 10.1107/S1600536811039250/hb6411Isup2.hkl

Supplementary material file. DOI: 10.1107/S1600536811039250/hb6411Isup3.cml

Additional supplementary materials:  crystallographic information; 3D view; checkCIF report

## Figures and Tables

**Table 1:** Hydrogen-bond geometry (Å, °) *Cg*2 and *Cg*3 are the centroids of the C3–C8 and C11–C16 rings, respectively.

*D*—H⋯*A*	*D*—H	H⋯*A*	*D*⋯*A*	*D*—H⋯*A*
C6—H6⋯*Cg*3^i^	0.95	2.85	3.719 (1)	152
C9—H9*A*⋯*Cg*2^ii^	0.98	2.94	3.799 (2)	147
C13—H13⋯*Cg*2^iii^	0.95	2.77	3.537 (2)	139

Symmetry codes: (i) 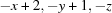; (ii) 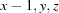; (iii) 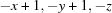.

**Fig. 1.**
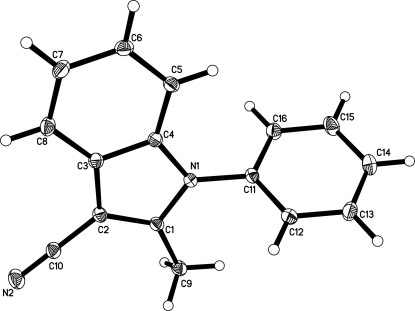
The molecular structure of (I) with 50% probability displacement ellipsoids.

## supplementary crystallographic information

### Comment

Indoles are an important compound possessing pharmaceutical properties. Extensive investigation on the crystal structures of indoles helps disclose their structure-activity relationship. For continuing our reseach, herein, we reported the crystal structure of the title indole derivative, (I).

In the molecular structure (Fig. 1), the components of the indole ring system are almost coplanar with a dihedral angle of 0.89 (7)° between its pyrrole part and benzene part. The indole ring forms an angle of 64.92 (5)° with the benzene ring.

In the molecular packing, π-π stacking and C—H···π interactions were observed, helping solidify the packing.

### Experimental

The title compound was prepared according to the method of the literature (Du *et al.*, 2006). Colourless prisms of (I) were grown from a mixture of ethyl actate and petroleum ether.

### Refinement

All H atoms were positioned geometrically (C—H = 0.95 and 0.98 Å)and refined as riding with *U*_iso_(H) = 1.2*U*_eq_(CH) or 1.5*U*_eq_(CH_3_).

### Crystal data

**Table:**

C_16_H_12_N_2_	*Z* = 2
*M**_r_* = 232.28	*F*(000) = 244
Triclinic, *P*1	*D*_x_ = 1.287 Mg m^−^^3^
Hall symbol: -P 1	Mo *K*α radiation, λ = 0.71075 Å
*a* = 6.3610 (5) Å	Cell parameters from 2709 reflections
*b* = 9.497 (1) Å	θ = 2.1–33.5°
*c* = 11.0210 (12) Å	µ = 0.08 mm^−^^1^
α = 65.97 (2)°	*T* = 113 K
β = 80.52 (2)°	Prism, colorless
γ = 88.13 (2)°	0.26 × 0.24 × 0.06 mm
*V* = 599.34 (14) Å^3^	

### Data collection

**Table:**

Rigaku Saturn724 CCD diffractometer	3494 independent reflections
Radiation source: rotating anode	2309 reflections with *I* > 2σ(*I*)
multilayer	*R*_int_ = 0.040
Detector resolution: 14.222 pixels mm^-1^	θ_max_ = 30.0°, θ_min_ = 2.1°
ω scans	*h* = −8→8
Absorption correction: multi-scan (*CrystalClear*-SM Expert; Rigaku, 2009)	*k* = −13→13
*T*_min_ = 0.980, *T*_max_ = 0.995	*l* = −15→15
11425 measured reflections	

### Refinement

**Table:**

Refinement on *F*^2^	Primary atom site location: structure-invariant direct methods
Least-squares matrix: full	Secondary atom site location: difference Fourier map
*R*[*F*^2^ > 2σ(*F*^2^)] = 0.044	Hydrogen site location: inferred from neighbouring sites
*wR*(*F*^2^) = 0.120	H-atom parameters constrained
*S* = 0.98	*w* = 1/[σ^2^(*F*_o_^2^) + (0.0674*P*)^2^] where *P* = (*F*_o_^2^ + 2*F*_c_^2^)/3
3494 reflections	(Δ/σ)_max_ = 0.002
164 parameters	Δρ_max_ = 0.45 e Å^−^^3^
0 restraints	Δρ_min_ = −0.29 e Å^−^^3^

### Special details

**Table:**

Geometry. All e.s.d.'s (except the e.s.d. in the dihedral angle between two l.s. planes) are estimated using the full covariance matrix. The cell e.s.d.'s are taken into account individually in the estimation of e.s.d.'s in distances, angles and torsion angles; correlations between e.s.d.'s in cell parameters are only used when they are defined by crystal symmetry. An approximate (isotropic) treatment of cell e.s.d.'s is used for estimating e.s.d.'s involving l.s. planes.
Refinement. Refinement of *F*^2^ against ALL reflections. The weighted *R*-factor *wR* and goodness of fit *S* are based on *F*^2^, conventional *R*-factors *R* are based on *F*, with *F* set to zero for negative *F*^2^. The threshold expression of *F*^2^ > σ(*F*^2^) is used only for calculating *R*-factors(gt) *etc*. and is not relevant to the choice of reflections for refinement. *R*-factors based on *F*^2^ are statistically about twice as large as those based on *F*, and *R*- factors based on ALL data will be even larger.

### Fractional atomic coordinates and isotropic or equivalent isotropic displacement parameters (Å^2^)

**Table:**

	*x*	*y*	*z*	*U*_iso_*/*U*_eq_	
N1	0.44315 (14)	0.77239 (10)	0.77878 (9)	0.0185 (2)	
N2	0.81470 (18)	0.84332 (13)	0.34779 (11)	0.0345 (3)	
C1	0.60803 (17)	0.82767 (13)	0.67167 (11)	0.0194 (2)	
C2	0.56979 (18)	0.77831 (13)	0.57514 (11)	0.0209 (2)	
C3	0.37200 (18)	0.68851 (13)	0.62387 (11)	0.0202 (2)	
C4	0.29646 (17)	0.68823 (12)	0.75088 (11)	0.0187 (2)	
C5	0.10695 (18)	0.61245 (13)	0.82939 (11)	0.0216 (2)	
H5	0.0582	0.6142	0.9150	0.026*	
C6	−0.00823 (18)	0.53441 (14)	0.77872 (12)	0.0249 (3)	
H6	−0.1391	0.4820	0.8299	0.030*	
C7	0.06551 (19)	0.53158 (14)	0.65265 (12)	0.0275 (3)	
H7	−0.0158	0.4763	0.6202	0.033*	
C8	0.2539 (2)	0.60756 (14)	0.57463 (12)	0.0257 (3)	
H8	0.3024	0.6049	0.4893	0.031*	
C9	0.78658 (18)	0.92844 (14)	0.66577 (12)	0.0246 (3)	
H9A	0.9227	0.8851	0.6449	0.030*	
H9B	0.7777	0.9348	0.7530	0.030*	
H9C	0.7772	1.0319	0.5955	0.030*	
C10	0.70487 (19)	0.81451 (14)	0.44957 (12)	0.0240 (3)	
C11	0.42676 (17)	0.78451 (12)	0.90546 (11)	0.0192 (2)	
C12	0.57306 (19)	0.71355 (14)	0.98980 (12)	0.0248 (3)	
H12	0.6857	0.6588	0.9629	0.030*	
C13	0.5548 (2)	0.72252 (14)	1.11344 (12)	0.0285 (3)	
H13	0.6578	0.6773	1.1701	0.034*	
C14	0.3862 (2)	0.79746 (14)	1.15407 (12)	0.0293 (3)	
H14	0.3723	0.8027	1.2393	0.035*	
C15	0.2379 (2)	0.86476 (15)	1.07108 (12)	0.0313 (3)	
H15	0.1207	0.9144	1.1002	0.038*	
C16	0.25896 (19)	0.86032 (14)	0.94531 (12)	0.0261 (3)	
H16	0.1589	0.9090	0.8873	0.031*	

### Atomic displacement parameters (Å^2^)

**Table:**

	*U*^11^	*U*^22^	*U*^33^	*U*^12^	*U*^13^	*U*^23^
N1	0.0180 (5)	0.0208 (5)	0.0170 (5)	0.0005 (4)	−0.0012 (3)	−0.0085 (4)
N2	0.0369 (6)	0.0412 (7)	0.0228 (5)	−0.0072 (5)	0.0039 (4)	−0.0131 (5)
C1	0.0185 (5)	0.0204 (5)	0.0174 (5)	0.0010 (4)	−0.0014 (4)	−0.0064 (4)
C2	0.0217 (6)	0.0224 (5)	0.0162 (5)	−0.0005 (4)	−0.0008 (4)	−0.0063 (4)
C3	0.0207 (6)	0.0209 (5)	0.0168 (5)	0.0004 (4)	−0.0029 (4)	−0.0054 (4)
C4	0.0187 (5)	0.0191 (5)	0.0177 (5)	0.0016 (4)	−0.0038 (4)	−0.0066 (4)
C5	0.0198 (6)	0.0236 (6)	0.0190 (5)	0.0003 (4)	−0.0006 (4)	−0.0071 (4)
C6	0.0198 (6)	0.0270 (6)	0.0247 (6)	−0.0036 (5)	−0.0030 (5)	−0.0072 (5)
C7	0.0279 (6)	0.0304 (6)	0.0253 (6)	−0.0051 (5)	−0.0080 (5)	−0.0108 (5)
C8	0.0306 (7)	0.0289 (6)	0.0178 (6)	−0.0019 (5)	−0.0056 (5)	−0.0089 (5)
C9	0.0235 (6)	0.0259 (6)	0.0228 (6)	−0.0043 (5)	0.0005 (4)	−0.0096 (5)
C10	0.0253 (6)	0.0261 (6)	0.0191 (6)	−0.0029 (5)	−0.0018 (4)	−0.0082 (5)
C11	0.0211 (5)	0.0191 (5)	0.0167 (5)	−0.0013 (4)	−0.0007 (4)	−0.0072 (4)
C12	0.0223 (6)	0.0291 (6)	0.0244 (6)	0.0048 (5)	−0.0032 (5)	−0.0127 (5)
C13	0.0323 (7)	0.0331 (7)	0.0220 (6)	0.0037 (5)	−0.0097 (5)	−0.0116 (5)
C14	0.0408 (8)	0.0278 (6)	0.0209 (6)	0.0001 (5)	−0.0030 (5)	−0.0121 (5)
C15	0.0363 (7)	0.0327 (7)	0.0267 (7)	0.0101 (6)	−0.0012 (5)	−0.0162 (5)
C16	0.0274 (6)	0.0287 (6)	0.0240 (6)	0.0086 (5)	−0.0059 (5)	−0.0123 (5)

### Geometric parameters (Å, °)

**Table:**

N1—C1	1.3757 (14)	C7—H7	0.9500
N1—C4	1.3955 (14)	C8—H8	0.9500
N1—C11	1.4337 (13)	C9—H9A	0.9800
N2—C10	1.1514 (15)	C9—H9B	0.9800
C1—C2	1.3819 (16)	C9—H9C	0.9800
C1—C9	1.4853 (16)	C11—C16	1.3797 (16)
C2—C10	1.4183 (16)	C11—C12	1.3849 (16)
C2—C3	1.4398 (17)	C12—C13	1.3854 (16)
C3—C8	1.4006 (16)	C12—H12	0.9500
C3—C4	1.4020 (15)	C13—C14	1.3815 (17)
C4—C5	1.3877 (16)	C13—H13	0.9500
C5—C6	1.3804 (16)	C14—C15	1.3797 (17)
C5—H5	0.9500	C14—H14	0.9500
C6—C7	1.4021 (17)	C15—C16	1.3881 (16)
C6—H6	0.9500	C15—H15	0.9500
C7—C8	1.3813 (17)	C16—H16	0.9500
			
C1—N1—C4	109.22 (9)	C3—C8—H8	120.7
C1—N1—C11	127.12 (9)	C1—C9—H9A	109.5
C4—N1—C11	123.45 (9)	C1—C9—H9B	109.5
N1—C1—C2	108.27 (10)	H9A—C9—H9B	109.5
N1—C1—C9	123.13 (10)	C1—C9—H9C	109.5
C2—C1—C9	128.56 (10)	H9A—C9—H9C	109.5
C1—C2—C10	124.37 (11)	H9B—C9—H9C	109.5
C1—C2—C3	108.35 (10)	N2—C10—C2	179.74 (14)
C10—C2—C3	127.28 (11)	C16—C11—C12	120.47 (10)
C8—C3—C4	119.01 (11)	C16—C11—N1	119.62 (10)
C8—C3—C2	135.08 (11)	C12—C11—N1	119.82 (10)
C4—C3—C2	105.90 (10)	C11—C12—C13	119.86 (11)
C5—C4—N1	129.20 (10)	C11—C12—H12	120.1
C5—C4—C3	122.53 (10)	C13—C12—H12	120.1
N1—C4—C3	108.27 (10)	C14—C13—C12	119.80 (11)
C6—C5—C4	117.64 (11)	C14—C13—H13	120.1
C6—C5—H5	121.2	C12—C13—H13	120.1
C4—C5—H5	121.2	C15—C14—C13	120.11 (11)
C5—C6—C7	120.81 (11)	C15—C14—H14	119.9
C5—C6—H6	119.6	C13—C14—H14	119.9
C7—C6—H6	119.6	C14—C15—C16	120.38 (11)
C8—C7—C6	121.36 (11)	C14—C15—H15	119.8
C8—C7—H7	119.3	C16—C15—H15	119.8
C6—C7—H7	119.3	C11—C16—C15	119.33 (11)
C7—C8—C3	118.63 (11)	C11—C16—H16	120.3
C7—C8—H8	120.7	C15—C16—H16	120.3
			
C4—N1—C1—C2	−0.61 (12)	C3—C4—C5—C6	0.38 (17)
C11—N1—C1—C2	174.22 (9)	C4—C5—C6—C7	0.39 (17)
C4—N1—C1—C9	177.02 (10)	C5—C6—C7—C8	−0.60 (18)
C11—N1—C1—C9	−8.15 (16)	C6—C7—C8—C3	0.03 (18)
N1—C1—C2—C10	179.25 (10)	C4—C3—C8—C7	0.71 (17)
C9—C1—C2—C10	1.79 (19)	C2—C3—C8—C7	179.55 (12)
N1—C1—C2—C3	0.15 (12)	C1—C2—C10—N2	113 (37)
C9—C1—C2—C3	−177.31 (11)	C3—C2—C10—N2	−68 (37)
C1—C2—C3—C8	−178.58 (13)	C1—N1—C11—C16	119.92 (13)
C10—C2—C3—C8	2.4 (2)	C4—N1—C11—C16	−65.94 (14)
C1—C2—C3—C4	0.36 (12)	C1—N1—C11—C12	−63.31 (15)
C10—C2—C3—C4	−178.71 (11)	C4—N1—C11—C12	110.84 (13)
C1—N1—C4—C5	−179.85 (11)	C16—C11—C12—C13	−1.93 (18)
C11—N1—C4—C5	5.09 (17)	N1—C11—C12—C13	−178.67 (10)
C1—N1—C4—C3	0.85 (12)	C11—C12—C13—C14	2.34 (19)
C11—N1—C4—C3	−174.22 (9)	C12—C13—C14—C15	−0.77 (19)
C8—C3—C4—C5	−0.94 (16)	C13—C14—C15—C16	−1.2 (2)
C2—C3—C4—C5	179.91 (10)	C12—C11—C16—C15	−0.06 (18)
C8—C3—C4—N1	178.42 (10)	N1—C11—C16—C15	176.69 (10)
C2—C3—C4—N1	−0.73 (12)	C14—C15—C16—C11	1.64 (19)
N1—C4—C5—C6	−178.84 (10)		

### Hydrogen-bond geometry (Å, °)

**Table:**

Cg2 and Cg3 are the centroids of the C3–C8 and C11–C16 rings, respectively.

**Table:**

*D*—H···*A*	*D*—H	H···*A*	*D*···*A*	*D*—H···*A*
C6—H6···Cg3^i^	0.95	2.85	3.719 (1)	152
C9—H9A···Cg2^ii^	0.98	2.94	3.799 (2)	147
C13—H13···Cg2^iii^	0.95	2.77	3.537 (2)	139

Symmetry codes: (i) −*x*+2, −*y*+1, −*z*; (ii) *x*−1, *y*, *z*; (iii) −*x*+1, −*y*+1, −*z*.
